# Balancing the Expression and Production of a Heterodimeric Protein: Recombinant Agkisacutacin as a Novel Antithrombotic Drug Candidate

**DOI:** 10.1038/srep11730

**Published:** 2015-07-06

**Authors:** Yugang Guo, Jing Wu, Hao Jia, Wei Chen, Changsheng Shao, Lei Zhao, Jiajia Ma, Rui Li, Yongjun Zhong, Fang Fang, Dong Wang, Jie Sun, Fang Qian, Xiangrong Dai, Guohui Zhang, Zhigang Tian, Benjamin Xiaoyi Li, Weihua Xiao

**Affiliations:** 1 The CAS Key Laboratory of Innate Immunity and Chronic Disease, Innovation Center for Cell Biology, School of Life Sciences, University of Science and Technology of China, Hefei, China; 2 Hefei National Laboratory for Physical Sciences at Microscale, Engineering Technology Research Center of Biotechnology Drugs, Anhui Province, University of Science and Technology of China, Hefei, China; 3 Anhui Engineering Research Center of Recombinant Protein Pharmaceutical Biotechnology, Institute of advanced technology, University of Science and Technology of China, Hefei, China; 4 Zhaoke Pharmaceutical (Hefei) Co. Ltd., Hefei, Anhui, China; 5 Lee’s Pharmaceutical Holdings Limited, Shatin, Hong Kong, China

## Abstract

Agkisacucetin extracted from the venom of *Agkistrodon acutus* has been demonstrated to be a promising antithrombotic drug candidate in clinical studies due to its function as a novel platelet membrane glycoprotein (GP) Ib inhibitor. Agkisacucetin is a heterodimeric protein composed of α- and β-subunits with seven disulphide bonds. Both subunits form inactive homodimeric products, which cause difficulties for recombinant production. In this study, Agkisacucetin α- and β-subunits were inserted sequentially into the chromosome of *Pichia pastoris* at the mutant histidinol dehydrogenase gene and ribosomal DNA repeat sites, respectively. By optimizing the gene copies and productivity of each subunit by drug screening, we successfully obtained a recombinant strain with balanced expression of the two subunits. Using this strain, a yield greater than 100 mg/L recombinant Agkisacucetin in fed-batch fermentation was reached. The recombinant Agkisacucetin possessed extremely similar binding affinity to recombinant GPIb and human platelets in *in vitro* assays, and its ristocetin-induced platelet aggregation activity *ex vivo* was identical to that of the extracted native Agkisacucetin, demonstrating that the yeast-derived Agkisacucetin could be an effective alternative to native Agkisacucetin. Moreover, this study provides an effective strategy for balancing the expression and production of heterodimeric proteins in *P. pastoris*.

Cardio-cerebral vascular diseases, particularly thrombosis, remain the most serious life-threatening diseases^1^. The development of safe and effective antithrombotic drugs without bleeding as a side effect is urgently needed. Platelet adhesion mediated by an interaction between platelet membrane glycoprotein (GP) Ib and von Willebrand factor (vWF), known as primary haemostasis, facilitates platelet aggregation mediated by GPIIb/IIIa complexes to promote thrombus formation^2^. Because platelet adhesion is one of the initial steps in the process of thrombogenesis, inhibition of platelet adhesion is considered an effective strategy to inhibit thrombosis and to prevent infarction with limited impact on the overall coagulation system^2^. GPIb is one of the key factors that mediate platelet adhesion under high shear conditions and an important target for new antiplatelet drug development^2^.

Snake venom Agkisacucetin (trade name Anfibatide) from *Agkistrodon acutus (A. acutus*) is a novel promising antiplatelet drug candidate that targets GPIb^3^,^4^,^5^. Agkisacucetin, which is a C-type-like lectin protein (CLP), binds to GPIb to prevent its binding to vWF, thus inhibiting platelet adhesion and aggregation^6^,^7^. Agkisacucetin markedly inhibited thrombus formation *in vivo* in animal models of thrombosis. More importantly, Agkisacucetin did not significantly cause platelet activation and bleeding in murine tests, which, together with other evidence, suggests that it has additional effects beyond its inhibitory role in the GPIb-vWF interaction^4^. Phase IIb clinical studies have recently produced encouraging results for the treatment of thrombosis with biochemically extracted natural Agkisacucetin (nAgkisacucetin) by Zhaoke Pharmaceutical (Hefei) Co. Ltd. (X. R. Dai, personal communication, 2014). However, nAgkisacucetin production from snake venom faces considerable challenges, including difficulties in the quality control of raw snake venom, the limit of natural resources and the risk of microbial contamination from snake venom.

Developing recombinant Agkisacucetin (rAgkisacucetin) has been difficult because of its characteristics and native structure. Agkisacucetin is a heterodimeric protein of 29 kDa that is composed of α- and β-subunits^7^. Three intramolecular disulphide bonds are present in each subunit, and one intermolecular disulphide bridge exists between the α- and β-subunits. Additionally, an oxidized sulphhydryl group is present at the N-terminus of the β-subunit in Agkisacucetin^7^. This native structure causes unusual difficulties for recombinant production, particularly for obtaining high-yield biologically active products in large-scale production. Previous attempts to express rAgkisacucetin in CHO cells in our laboratory failed due to severely unbalanced expression of the α- and β-subunits.

In this study, we successfully developed a simple strategy for balancing the expression of rAgkisacucetin in *P. pastoris*. The established recombinant *P. pastoris* strain yielded greater than 100 mg/L biologically active rAgkisacucetin from the culture medium in a 14 L high-density fermentation process. Through downstream filtration in combination with common chromatography, greater than 95% purity was achieved for the final products. rAgkisacucetin has the same binding affinity to the recombinant GPIb and human platelets as nAgkisacucetin. An *ex vivo* assay with human peripheral blood showed platelet adhesion inhibitory activity was extremely similar to that of nAgkisacucetin. This study established an effective strategy for balancing the expression and production of rAgkisacucetin, which could be employed for the production of other heteromultimeric proteins.

## Results

### Construction and screening of balanced Agkisacutacin expression strains

**Figure 1:**
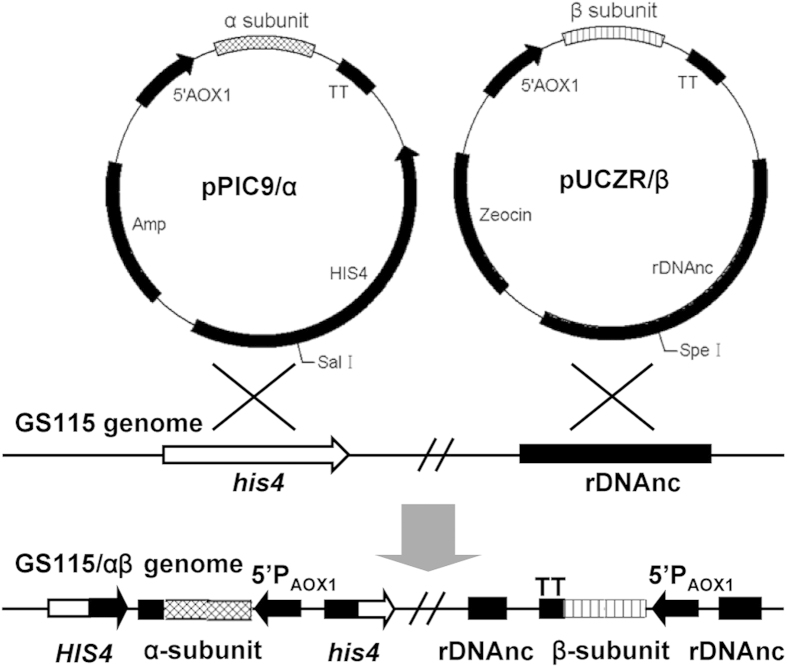
Strategy for the construction of rAgkisacutacin expression strains. This schematic map represents the constructed expression vectors for the Agkisacutacin α- and β-subunits, designated pPIC9/α and pUCZR/β, respectively, in which, *his4* and rDNA non-coding sequences allow the vector to be inserted into the corresponding sites in the genome of strain GS115 through homologous recombination. The pUCZR vector was constructed by introducing a non-coding rDNA (indicated as rDNAnc) sequence into the pPICZα vector. The expression of both α- and β-subunits was under the control of the AOX1 promoter.

**Figure 2:**
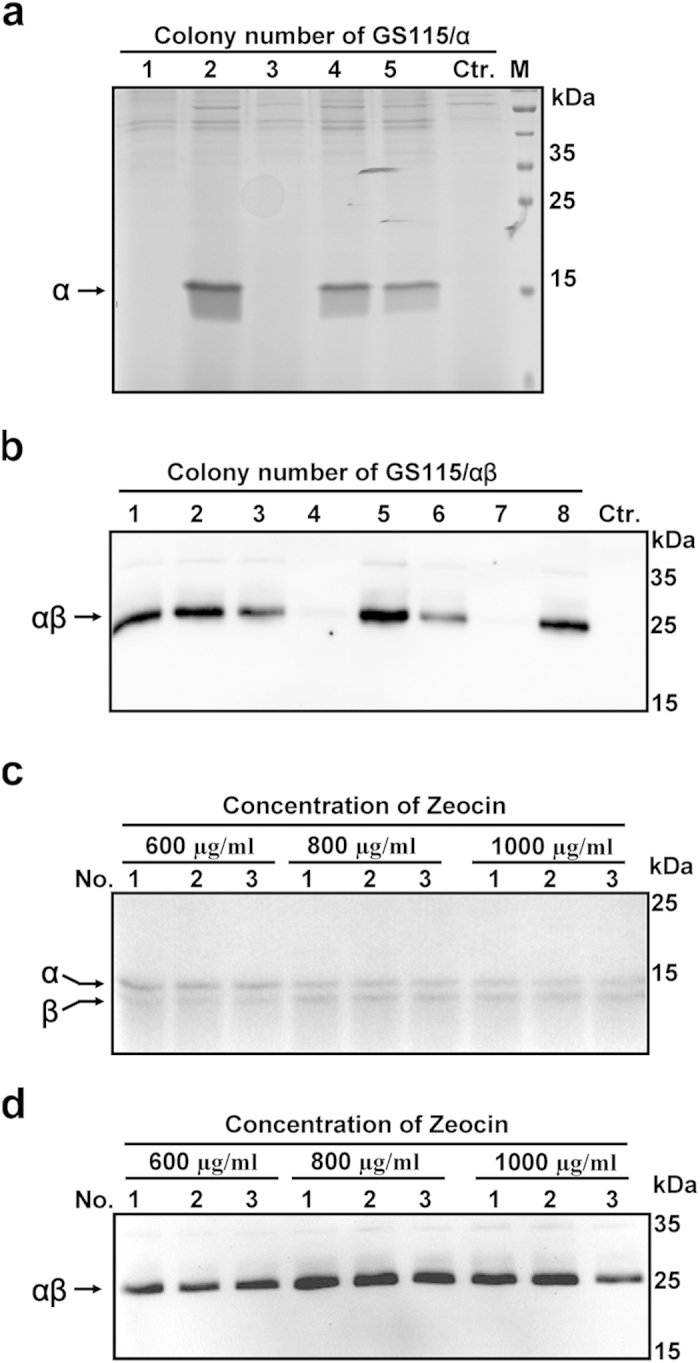
Establishing balanced expression strains for rAgkisacutacin subunits. (**a**) The expression of the α-subunit in transformants, designated GS115/α, was examined by 15% SDS-PAGE under reducing conditions, and colony with the highest expression was selected for further transformation with the β-subunit expressing vector. (**b**) The expression of the αβ heterodimer from the transformants, designated GS115/αβ, was examined by Western blot, and the colony with the highest expression was used for further drug screening. Ctr.: colony transformed with empty vectors. The expression of the α- and β-subunits from screened colonies with various doses of Zeocin was examined by SDS-PAGE under reducing conditions (**c**) or by Western blot under non-reducing conditions (**d**). No.: colony number.

The coding sequence of the α- or β-subunit of Agkisacutacin was inserted into the *Xho* I- *Not* I sites of the pPIC9 or pUCZR vectors, and the resulting constructs were designated pPIC9/α or pUCZR/β, respectively (Fig. 1). The pPIC9/α and pUCZR/β vectors contain the functional histidinol dehydrogenase (*HIS4)* and rDNA repeat genes, respectively, for homologous recombination into the genome of *P. pastoris*. Both the α- and β-subunit coding genes were fused with the *Saccharomyces cerevisiae* α-mating factor signal sequence and placed under the regulation of an AOX1 promoter. To generate the balanced expression strain, the Agkisacutacin α-subunit expressing plasmid was transformed into *P. pastoris* strain GS115 and selected using histidine-deficient minimal dextrose (MD) plates. The colony with the highest α-subunit expression was selected by SDS-PAGE (Fig. 2a) and subjected to further transformation with the β-subunit-expression vector pUCZR/β. The α- and β-subunit co-expression colonies GS115/αβ were screened using histidine-deficient MD plates containing 600 μg/ml Zeocin, and the expression of αβ heterodimeric proteins was monitored by Western blot using anti-Agkisacutacin monoclonal antibody 1B9 (Fig. 2b). The colony with the highest relative expression was selected for further balanced expression screening by stepwise increases in the drug concentration with 600 μg/ml, 800 μg/ml and 1000 μg/ml Zeocin on histidine-deficient MD plates. The expression of each subunit individually and the αβ heterodimer in the resulting colonies was determined by SDS-PAGE under reducing (Fig. 2c) and non-reducing (Fig. 2d) conditions, respectively. The expression of the α- and β-subunits gradually shifted from more α-subunit than β-subunit to more β-subunit than α-subunit when the concentration of Zeocin was increased from 600 μg/ml to 1000 μg/ml (Fig. 2c). With 800 μg/ml Zeocin, the expression of the α- and β-subunits reached a balance, with nearly equal amounts (Fig. 2c). The highest expression of αβ heterodimer (Fig. 2d) was also found in colonies with balanced expression of the α- and β-subunits. The strategy of combining the mutant histidinol dehydrogenase gene (*his4*) and rDNA repeat loci for homologous recombination and stepwise drug screening achieved a successful result for balancing the expression of rAgkisacutacin in *P. pastoris*.

### Expression condition optimization

**Figure 3:**
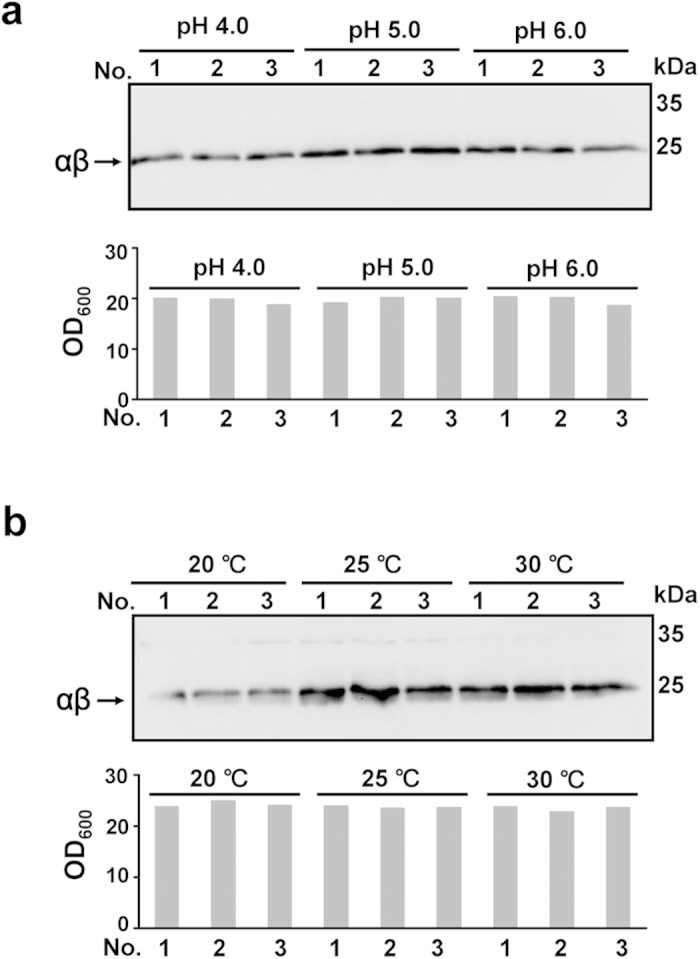
Optimization of culture conditions for rAgkisacutacin expression in flasks. Protein expression (upper panel) and the corresponding biomass (lower panel) in shake flasks at indicated pH (**a**) or temperatures (**b**) were examined. Three individual colonies were tested for each condition.

The optimal culture conditions, including pH and temperature, for protein expression were determined using shake flasks. The best condition for rAgkisacutacin expression was at pH 5.0 (Fig. 3a). The optimal temperature range was from 25 °C ~ 30 °C (Fig. 3b). Neither pH nor temperature had a significant effect on the biomass in the cultures (Fig. 3a,b).

### Pilot-scale fermentation of rAgkisacutacin

**Figure 4:**
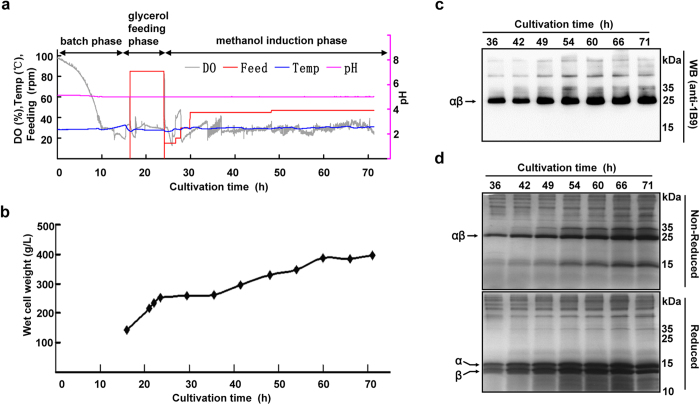
Pilot-scale fermentation of rAgkisacutacin. (**a**) The plotted parameters show the DO, pH, temperature and the feeding speed for glycerol and methanol during a typical three-step fermentation process with a 14-L New Brunswick BioFlo 115 fermentor. (**b**) The growth rate was measured and presented as WCW during the fermentation process. rAgkisacutacin expression during the fermentation was examined by Western blot with anti-Agkisacutacin antibody 1B9 (**c**) and by SDS-PAGE stained with Coomassie blue (**d**) under reducing (lower panel) and non-reducing (upper panel) conditions.

The typical fermentation process (Fig. 4a) was composed of three steps: a batch phase, glycerol feeding phase and methanol induction phase. During the batch phase, the yeast seeds were cultured with BMGY medium containing 4% glycerol (pH 5.0) and controlled at 28 °C. The batch phase usually lasted 17–18 h and ended when the wet cell weight (WCW) reached 140 g/L and a sharp dissolved oxygen (DO) spike occurred, indicating the depletion of glycerol in the culture medium. During the glycerol feeding phase, 50% glycerol supplemented with 12 ml/L PTM1 solution was supplied through feeding, and DO was controlled at 30% by limiting airflow. The temperature was maintained at 28 °C, and pH remained at 5.0. This process usually lasted 7–8 h and ended when the WCW reached approximately 250 g/L. After feeding stopped and a DO spike was observed, the methanol induction phase was started by a stepwise increase in the methanol feeding rate (100% methanol with 12 ml/L PTM1 salts) from 21.6 ml/min to 64.8 ml/min over 4–5 h. The DO was restricted to approximately 30% by limiting the supply of methanol and oxygen (Fig. 4a). The methanol induction phase lasted 45 h and ended when the yeast WCW reached approximately 400 g/L (Fig. 4b). The accumulated rAgkisacutacin increased progressively during the first 35 h of methanol induction; however, prolonged cultivation led to either stagnation or a decline in both cell growth (Fig. 4b) and rAgkisacutacin production (Fig. 4c). Moreover, the levels of the α- and β-subunits remained equal throughout the fermentation process (Fig. 4d).

### Purification and characterization of rAgkisacutacin

**Figure 5:**
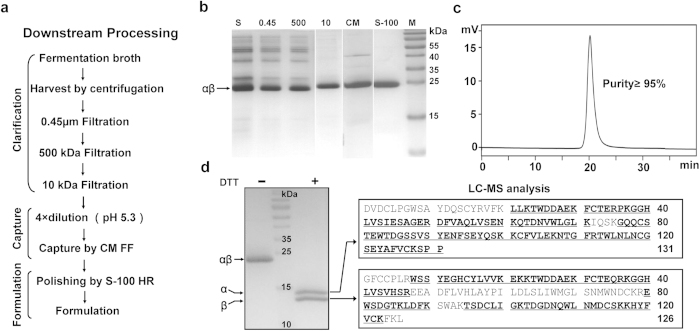
Downstream processing and characterization of rAgkisacutacin. (**a**) Schematic presentation of the workflow for downstream processing. The culture supernatant was collected and clarified by centrifugation and stepwise filtration. After diluting the sample to adjust the pH and salt concentration, rAgkisacutacin protein was captured by CM FF and further purified using S-100 HR chromatography. (**b**) The samples from each step during the purification processes were monitored by 15% SDS-PAGE. S: the supernatant from centrifugation; 0.45/500/10 FT: the flow-through of filtration through 0.45 μm/500 kDa/10 kDa hollow-fibre membranes; CM: CM FF column; S-100: S-100 HR column; M: pre-stained protein molecular marker. (**c**) The purity of rAgkisacutacin was analysed by SCE. (**d**) Approximately 20 μg of final purified rAgkisacutacin was analysed by 15% SDS-PAGE under reducing and non-reducing conditions as indicated and was visualized by Coomassie blue staining. The bands corresponding to the α- and β-subunits were excised and digested for LC-MS analysis. The amino acid sequences of the α- and β-subunits are listed, and the detected peptides from LC-MS are underlined and bolded.

The downstream processing of rAgkisacutacin fermentation was composed of three steps (Fig. 5a): clarification of the broth by centrifugation and filtration, capturing rAgkisacutacin by ion-exchange chromatography, and formulation by molecular-exclusion chromatography. The products from each step were monitored by SDS-PAGE (Fig. 5b). Using this process, the final products surpassed 95% purity as determined by HPLC (Fig. 5c). Additionally, both the α- and β-subunits of rAgkisacutacin were characterized by LC-MS, which showed the sequence coverage of most of the proteins and which confirmed that the sequence of rAgkisacutacin exactly matched that of nAgkisacutacin (Fig. 5d). Thus, an effective downstream purification process was established for rAgkisacutacin production.

### Biological activity of rAgkisacutacin

**Figure 6:**
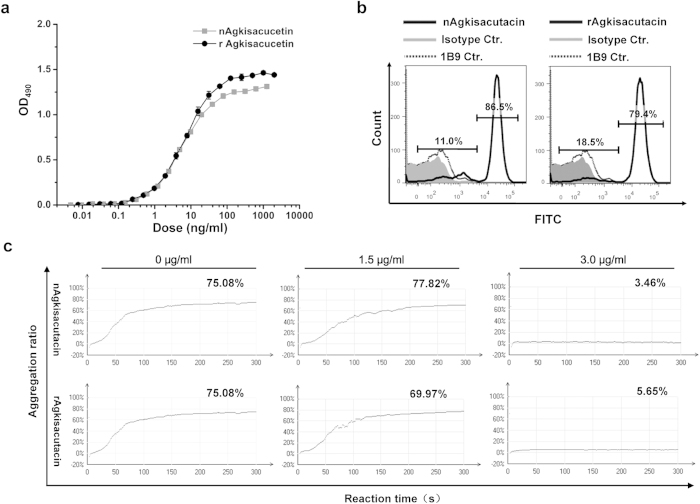
Determination of the biological activity of rAgkisacutacin. (**a**) rGPIb binding assays were performed to measure the relative binding affinities of rAgkisacutacin and nAgkisacutacin. (**b**) *Ex vivo* binding assays with fluorescently labelled nAgkisacutacin or rAgkisacutacin to platelets from human blood were measured by flow cytometry; nAgkisacutacin/rAgkisacutacin: nAgkisacutacin/rAgkisacutacin+1B9+FITC-anti-mouse IgG; Isotype Ctr.: nAgkisacutacin/rAgkisacutacin+1B9+isotype control; 1B9 Ctr.: 1B9+FITC-anti-mouse IgG. (**c**) Ristocetin-induced platelet aggregation activity for the indicated doses of rAgkisacutacin and nAgkisacutacin are presented; the 0 μg/ml group is the control.

The binding affinity of rAgkisacutacin to solid-phase rGPIb was determined and compared with nAgkisacutacin by ELISA (Fig. 6a). Both rAgkisacutacin and nAgkisacutacin bound to rGPIb in a concentration-dependent manner, with extremely similar half-maximal binding concentrations of 6.5 ± 0.1 ng/ml and 6.1 ± 0.5 ng/ml, respectively (Fig. 6a). The *ex vivo* FACS assay with human whole blood showed that the binding activities of rAgkisacutacin and nAgkisacutacin were 79.4% and 86.5%, respectively (Fig. 6b). Antiplatelet activity was measured by ristocetin-induced platelet aggregation assay. Both rAgkisacutacin and nAgkisacutacin showed dose-dependent antiplatelet activity and nearly complete reduction of aggregation with rates of 5.65% and 3.46%, respectively at the 3 μg/ml dose (Fig. 6c). These data consistently indicate that rAgkisacutacin possesses extremely similar antiplatelet activity to that of nAgkisacutacin.

## Discussion

Many bioactive molecules, such as human interleukin 12, therapeutic antibodies and recombinant factor VIII-Fc, are biologically active in their heterodimeric forms^8^,^9^,^10^. However, balancing the expression of each subunit of a heterodimeric protein at high efficacy remains a challenging task^11^,^12^,^13^,^14^. This study is the first report of the successful establishment of a balanced expression strain and pilot-scale production of functionally active rAgkisacutacin in *P. pastoris*.

A common challenge for the expression of heterodimeric proteins is that each subunit in a heterodimeric protein can form homodimers, which could act as a decoy for its active partners and interfere with the production rate and with downstream purification^15^. In this study, the two subunits of Agkisacutacin were inserted into different loci in the host genome at *his4* and rDNA repeats with distinct selectable markers of *his4* deficiency and Zeocin antibiotic to allow for the screening of colonies expressing each subunit individually or together. Another advantage of transforming each subunit into a different locus of the host genome is that each gene is expressed independently to avoid mutual interference of transcription and translation, which could explain why the strains with balanced expression of the rAgkisacutacin α- and β-subunits also had the highest levels of αβ heterodimer production (Fig. 2c,d). Given that the genome of *P. pastoris* contains 21 copies^16^ of rDNA at repeat loci and only a single copy of *his4*^17^, the frequency of homologous recombination under drug screening is much greater at the rDNA repeats than at the *his4* site. This difference in the frequency of homologous recombination between the rDNA repeat and *his4* loci is most likely the underlying mechanism for the gradual significant increase in expression of the β-subunit when the αβ co-expressing strains were screened with increasing concentrations of Zeocin (Fig. 2c), which is consistent with a recent report regarding the expression of opioids^18^.

**Figure 7:**
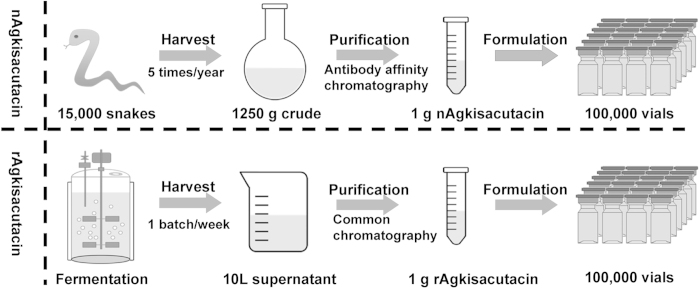
Schematic comparison of the manufacturing processes for recombinant and extracted natural Agkisacutacin.

The optimized fermentation parameters for rAgkisacutacin expression including pH and temperature were determined using shake flasks. The optimal conditions were pH 5.0 and 30 °C. Accordingly, a simple and robust pilot-scale fed-batch cultivation process was established and yielded approximately 1 g of final rAgkisacutacin product, which equals the total quantity of nAgkisacutacin in raw venom from 15,000 snakes per year (Fig. 7).

To clarify the supernatant and to eliminate protein contamination in the culture, multiple hollow-fibre membranes were utilised following liquid-solid separation by centrifugation. However, when testing a 10-kDa membrane for the collection and concentration of rAgkisacutacin according to its predicted molecular weight of 29 kDa, surprisingly, most of the contaminant proteins were removed, and rAgkisacucetin was found in the flow-through phase. The passage of Agkisacucetin through the 10-kDa membrane may be enhanced by its pencil-like shape, which is apparent in its crystal structure^7^. Thus, the 10-kDa membrane was utilised for filtration in the downstream process.

Agkisacutacin is a GPIb-binding protein that inhibits platelet adhesion by inhibiting the interaction between GPIb and vWF to block the clustering of GPIb^2^,^7^. In both the *in vitro* and *ex vivo* assays, rAgkisacutacin possessed functional characteristics similar to those of nAgkisacutacin. Notably, rAgkisacutacin retained approximately 94% of binding activity to rGPIb *in vitro* and 92% of binding activity to human platelets *ex vivo*. These results are much better than those of recombinant snake CLPs such as bothrojaracin and ACFI, with only 20% and 30% binding activity compared to their native counterparts, respectively^15^,^19^. Both rAgkisacutacin and nAgkisacutacin displayed excellent inhibition of ristocetin-induced platelet aggregation. The proper biological functions of many heterodimers rely on the correct heterodimerization of their subunits^15^. Thus, the improved potency of rAgkisacutacin might be due to the balanced expression of the α- and β-subunits, increasing the successful formation of heterodimers. These results suggest that *P. pastoris* is a promising system for balancing the expression of biologically active heterodimers in large scale for both structure-function studies and clinical research.

## Methods

### Ethics statement

Healthy human peripheral blood was obtained from the Blood Centre of Anhui Province (Hefei, China), and all participants provided written informed consent. All experiments were performed in accordance with the approved guidelines of the Ethics Committee of the University of Science and Technology of China.

### Strains, plasmids, antibodies and equipment

*P. pastoris* strain GS115, *E. coli* strain DH5α and expression vectors pPIC9 and pPICZα were purchased from Invitrogen (Life Technologies, Carlsbad, CA). Anti-Agkisacutacin monoclonal antibody 1B9 was obtained from Zhaoke Pharmaceutical (Hefei) Co. Ltd. as described previously.^3^ HPR-conjugated anti-mouse antibodies were purchased from Cell Signaling Technology (Beverly, MA). The 14 L fermentor (New Brunswick BioFlo 115) used in the pilot-scale fermentation process was obtained from Eppendorf (Enfield, CT). FlexStand Systems with 0.45 μm, 500 kDa and 10 kDa hollow-fibre membrane filtration cartridges were obtained from GE Healthcare (Piscataway, NJ), and the AKTA avant System with CM FF and S-100 HR columns was obtained from GE Healthcare (Uppsala, Sweden). The high-performance liquid chromatography (HPLC) system (LC-20AD) with a TSK3000SW 5 μm 250 mm × 4.6 mm column (Tosoh Co., Tokyo, Japan) was obtained from Shimadzu (Kyoto, Japan). The ELX800 microplate reader was obtained from Bio-Tek (Winooski, VT). The BD FACSVerse flow cytometer was obtained from BD BioSciences (Franklin Lakes, NJ). The analysis software FlowJo was obtained from Tree Star (San Carlos, CA).

### Vector construction, transformation and expression

Optimized coding sequences for the α- and β-subunits of Agkisacutacin were assembled by total gene synthesis (TaKaRa, Dalian, China) according to previously published amino acid sequences^7^. The coding sequence of the α-subunit of Agkisacutacin was cloned into *Xho* I and *Not* I restriction enzyme sites of the pPIC9 vector, resulting in pPIC9/α. The pUCZR vector was constructed in our lab by introducing an rDNA non-coding sequence into the *Bam*H I site of pPICZα for rDNA targeted gene integration. The coding sequence of the β-subunit of Agkisacutacin was cloned into *Xho* I and *Not* I restriction enzyme sites of the pUCZR vector, resulting in pUCZR/β. Both insertion sequences in pPIC9/α and pUCZR/β were confirmed by DNA sequencing. All of the restriction enzymes, primers, reagents and sequencing used in cloning were supplied by Sangon (Shanghai, China).

Vectors pPIC9/α and pUCZR/β were linearised using *Sal* I or *Spe* I digestion, respectively, and transformed into *P. pastoris* strain GS115 as described previously^20^. The transformants with pPIC9/α and pUCZR/β were screened on histidine-deficient MD plates with varying concentrations of Zeocin (Sigma, Ronkonkoma, NY). Culture conditions of the transformants were optimised in shake-flasks. The parameters included culture media pH (4.0, 5.0 and 6.0) and induction temperature (20 °C, 25 °C, and 30 °C) were performed in triplicate.

### Pilot-scale fermentation

Pilot-scale fermentation of rAgkisacutacin was performed according to *Pichia* fermentation process guidelines (Invitrogen, Life Technologies) and to a previous report with modifications^20^. Specifically, the inocula were cultured in a 1-L shake flask containing 200 ml BMGY media at 30 °C for 24 h and then transferred to a 14 L NBS BioFlo 115 Fermentor with 6 L BMGY media containing 4% glycerol. The culture temperature was maintained at 28 °C, and the pH was controlled at 5.0 with ammonium hydroxide. The rapid increase in DO suggested that glycerol had been consumed; thus, glycerol feeding was then initiated. When the glycerol feeding was stopped and a DO spike occurred, methanol (100% methanol with 12 ml/L PTM1 salts) solution was fed to the culture for induction. The DO value was controlled at approximately 30% by limited methanol feeding and oxygen supply during the remainder of fermentation.

### Purification of Agkisacutacin

The culture supernatant was separated, collected by centrifugation at 10,000 × *g* for 20 min and further sequentially clarified using a FlexStand System with 0.45 μm, 500 kDa and 10 kDa hollow-fibre membranes. The clarified supernatant was diluted 1:4 with low salt buffer (20 mM sodium phosphate buffer, pH 5.3), and the target proteins were captured using a 300-ml CM FF column on an AKTA avant System, and eluted with 30% buffer B (1 M NaCl, 20 mM sodium phosphate buffer, pH 5.3). The captured proteins were further purified with a S-100 HR column at a flow speed of 1 ml/min and analysed by SDS-PAGE. The purity of rAgkisacutacin was determined using size exclusion chromatography (SCE) with a TSK3000SW 5 μm 250 mm × 4.6 mm column on a Shimadzu HPLC System according to the manufacturer’s manual. LC-MS was performed as described previously^20^.

### Binding activity assay

The *in vitro* binding activity of Agkisacutacin to the rGPIb (expressed in CHO cells) was determined by sandwich ELISA according to a previous report with modifications.^15^ The 96-well plate was coated with 5 μg/ml rGPIb per well overnight at 4 °C and then washed twice with PBS containing 1% Tween-20 (PBST). After blocking with 300 μL assay buffer (0.5% BSA in PBST), serial dilutions of rAgkisacutacin and nAgkisacutacin were added to each well and incubated for 2 h at room temperature. The binding activity was quantified by measuring the absorbance at OD_490_ using an ELX800 microplate reader after incubation with anti-Agkisacutacin monoclonal antibody 1B9 (1:2000) and HRP-conjugated anti-mouse antibody (1:5000).

The *ex vivo* binding activity assay of Agkisacutacin to human platelets was performed using human whole peripheral blood from healthy adult volunteers. Briefly, 10 μg/ml rAgkisacutacin or nAgkisacutacin were incubated with 0.1 ml human blood for 30 min and then labelled with 1B9 followed by FITC-anti-mouse IgG or isotype control. The FITC fluoresce intensity was detected using a BD FACSVerse flow cytometer. The data were analysed using FlowJo.

### Ristocetin-induced platelet aggregation assay

Ristocetin-induced platelet aggregation assays were performed according to a previous report^21^. Briefly, platelet-rich plasma (PRP) and platelet-poor plasma (PPP) were isolated from human blood by centrifugation (3000 rpm) for 10 min at room temperature. The platelet density in PRP was adjusted to 250 × 10^9^/L (±10%) by adding the appropriate amount of PPP. rAgkisacutacin or nAgkisacutacin, together with 2 mg/ml ristocetin (ABP, London, UK), was added into the prepared PRP. Samples without Agkisacutacin were used as controls. The aggregations of platelets were automatically recorded by a platelet aggregometer (LBY-NJ4, Beijing, China). The platelet aggregation rate was defined as the maximal percent increase within the first 300 seconds.

## Additional Information

**How to cite this article**: Guo, Y. *et al.* Balancing the Expression and Production of a Heterodimeric Protein: Recombinant Agkisacutacin as a Novel Antithrombotic Drug Candidate. *Sci. Rep.* **5**, 11730; doi: 10.1038/srep11730 (2015).
